# Magnetically Induced Thermal Effects on Tobacco Mosaic Virus-Based Nanocomposites for a Programmed Disassembly of Protein Cages

**DOI:** 10.1021/acsabm.4c00634

**Published:** 2024-06-27

**Authors:** Ecem Tiryaki, Carla Álvarez-Leirós, Julia N. Majcherkiewicz, Paul L. Chariou, Melodie Maceira-Campos, Gustavo Bodelón, Nicole F. Steinmetz, Verónica Salgueiriño

**Affiliations:** † CINBIO, Universidade de Vigo, Vigo 36310, Spain; ‡ Department of Bioengineering, University of California San Diego, La Jolla, California 92093, United States; § Departamento de Biología Funcional y Ciencias de la Salud, Universidade de Vigo, Vigo 36310, Spain; ∥ Department of NanoEngineering, University of California San Diego, La Jolla, California 92093, United States; ⊥ Department of Radiology, University of California San Diego, La Jolla, California92093, United States; # Center for Nano-ImmunoEngineering, University of California San Diego, La Jolla, California92093, United States; ∇ Institute for Materials Discovery and Design, University of California San Diego, La Jolla, California92093, United States; ⊗ Departamento de Física Aplicada, Universidade de Vigo, Vigo 36310, Spain

**Keywords:** tobacco mosaic virus, magnetic nanoparticles, nanocomposites, magnetic hyperthermia, SAR values, protein cage disassembly, gene delivery

## Abstract

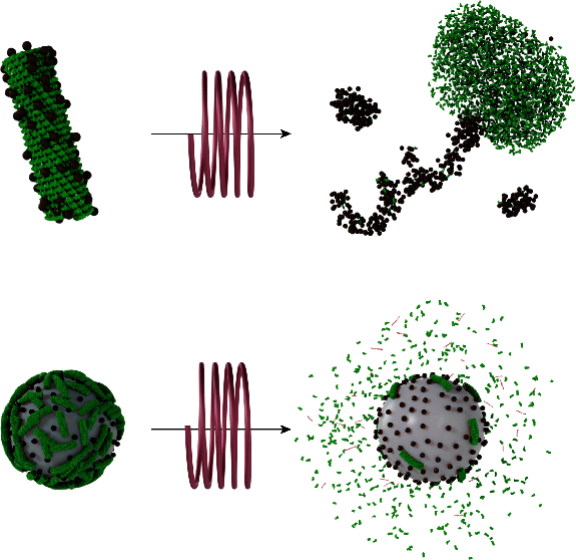

Protein cages are promising tools for the controlled delivery of therapeutics and imaging agents when endowed with programmable disassembly strategies. Here, we produced hybrid nanocomposites made of tobacco mosaic virus (TMV) and magnetic iron oxide nanoparticles (IONPs), designed to disrupt the viral protein cages using magnetically induced release of heat. We studied the effects of this magnetic hyperthermia on the programmable viral protein capsid disassembly using (1) elongated nanocomposites of TMV coated heterogeneously with magnetic iron oxide nanoparticles (TMV@IONPs) and (2) spherical nanocomposites of polystyrene (PS) on which we deposited presynthesized IONPs and TMV via layer-by-layer self-assembly (PS@IONPs/TMV). Notably, we found that the extent of the disassembly of the protein cages is contingent upon the specific absorption rate (SAR) of the magnetic nanoparticles, that is, the heating efficiency, and the relative position of the protein cage within the nanocomposite concerning the heating sources. This implies that the spatial arrangement of components within the hybrid nanostructure has a significant impact on the disassembly process. Understanding and optimizing this relationship will contribute to the critical spatiotemporal control for targeted drug and gene delivery using protein cages.

## Introduction

Nanobiotechnology has made significant strides in the development of programmable nanocarriers tailored for the targeted delivery of therapeutic cargoes or diagnostics. In this context, protein-based nanomaterials constitute an emerging class of highly attractive tools in biomedicine, which due to their diverse physicochemical properties can be assembled in different morphologies and can circumvent different physiological, extracellular, and intracellular barriers.^1^ In fact, as a bioengineering platform, they offer two key features, a polyvalent surface and an available volume for containment.^2^

Among these innovative protein-based nanosystems, viruses stand out as nanostructures with precisely defined shapes designed to protect and transport their genetic payload. These viral protein cages have demonstrated efficacy as cargo carriers,^3,4^ anticancer therapeutics,^5,6^ and vaccines.^7−9^ Moreover, native virus capsids possess unique assembly properties, rendering them attractive building blocks for bottom-up nanobioengineering solutions, particularly when combined with DNA.^10,11^ In particular, nanoparticles derived from tobacco mosaic virus (TMV) and cowpea chlorotic mottle virus (CCMV) have emerged as promising carriers for targeted drug delivery due to their nonpathogenic nature in humans and scalability in production.^12−15^ Plant viruses have also been investigated as gene delivery vectors, given that they lack the machinery necessary to navigate mammalian cells efficiently, hindering therefore trafficking, unpacking, and gene expression.

While a considerable amount of work is focused on creating robust protein nanocages,^16^ driven by diverse applications in medicine and biotechnology, the mechanisms for cage disruption remain unrestrained or with a vague control. More specifically, in the cases linked to drug delivery, there are limited methods for the subsequent opening for cargo release under explicit conditions. Some mechanisms of cage disruption associated with methods based on changes in pH,^17^ hydrostatic pressure,^18^ and chemicals present in the media^19^ have been reported, but few of them are triggered by external factors, such as light.^20^

When it comes to considering virus capsids, simulation studies examining the release of genomic material from virus capsids have underscored the importance of achieving a burst release of drug/genome content when utilizing protein cages.^21^ However, current techniques for this precise spatiotemporal control over the disassembly of these protein cages remain elusive. Consequently, we propose virus-based nanostructures equipped with a magnetic functionality, for a transformative targeted opening, enabled with guided movement,^22^ circumventing endocytosis,^23^ and the option of a rapid release of cargo by denaturing the protein cages using heat generated when exposed to an alternating magnetic field.^24,25^ With this into account, we report prototypes for the programmable disassembly of protein capsids from the TMV using magnetic iron oxide nanoparticles (IONPs) as actuators of magnetic hyperthermia. These proof-of-concept hybrid materials open up promising strategies for gene and drug delivery applications to maximize the therapeutic efficacy at the lowest possible therapeutic dose and in a programmed way, minimizing side effects.

## Experimental Section

### Chemicals

Iron(II) sulfate heptahydrate (FeSO_4_·7H_2_O, 99%), hydrazine hydrate (N_2_H_4_, 64%), iron(III) chloride hexahydrate (FeCl_3_·6H_2_O, ≥99%), tetramethylammonium hydroxide (TMAOH, 30 vol %), hydrochloric acid (HCl, 37 wt %), ammonium hydroxide (NH_4_OH, 28–30 wt %), poly(allylamine hydrochloride) MW ∼ 17,500 (PAH), poly(sodium 4-styrenesulfonate) MW ∼ 70,000 (PSS), sodium chloride (NaCl, 99%), and ethanol anhydrous were purchased from Sigma-Aldrich. Polystyrene spheres (PS, 500 nm, 100 mg mL^–1^) were provided by Thermo Fisher Scientific. Milli-Q water (18.2 MΩ cm resistance) was also applied. All chemicals were used without any further purification or treatment.

### Production and Characterization of TMV

TMV was propagated in *Nicotiana benthamiana* leaves and purified as previously described.^26^ Purified TMV was characterized using a combination of UV/vis spectroscopy, dynamic light scattering (DLS), size exclusion chromatography, and denaturing gel electrophoresis to confirm the integrity of the particles. The UV/vis spectrum of TMV was recorded using a NanoDrop spectrophotometer using the following parameters: ε (260 nm) = 3.0 mL mg^–1^ cm^–1^, molecular weight of TMV = 39.4 × 10^6^ g mol^–1^. TMV (200 μL, 1 mg mL^–1^) was eluted through a Superose 6 Increase column on the AKTA Explorer chromatography system (GE Healthcare) using a flow rate of 0.5 mL min^–1^ in 10 mM KP buffer (pH 7.0). The absorbance at 260 and 280 nm was recorded. The hydrodynamic *z*-average size of TMV (1 mg mL^–1^) was established in 10 mM phosphate buffer (KP) buffer using a Zetasizer Nano ZSP/ZEN5600 instrument (Malvern Panalytical). The TMV *z*-average size was calculated as the weighted mean of the intensity distribution. For analysis of the coat proteins, TMV was denatured at 95 °C for 5 min with 4× LDS loading dye. Samples were run on 12% SDS-PAGE precast gels in 1× morpholinepropanesulfonic acid (MOPS) buffer at 200 V and 120 mA for 40 min in the presence of SeeBlue Plus2 ladder size markers. Gels were imaged after staining for proteins using Coomassie Brilliant Blue (0.25% w/v)) with a FluorChem R imaging set to white light.

### Hybrid Nanocomposites of TMV Coated with Magnetic Iron Oxide Nanoparticles (TMV@IONPs)

Hybrid nanocomposites of TMV and IONPs were produced by depositing the iron oxide phase directly onto the virus; 100 μL of TMV (10 mg mL^–1^) was dispersed in 10 mL of ultrapure water and stirred. 200 μL of FeSO_4_ (50 mM) aqueous solution was added and left with the virus overnight under gentle agitation. 1 mL of hydrazine was added, stirred for 3 h, and then centrifuged (1200*g*, 10 min) to remove any unreacted species and redispersed in 10 mL of ultrapure water. Finally, the resuspended nanocomposites were immersed in a thermostatic bath at 60 °C for 15 min, washed by centrifugation, and redispersed in ultrapure water.

### Polystyrene (PS) Spheres Coated with Presynthesized Iron Oxide Nanoparticles and TMV (PS@IONPs/TMV)

To produce these nanocomposites, Massart-type presynthesized (Fe_3_O_4_/γ-Fe_2_O_3_) nanoparticles (IONPs) were employed.^27^ Accordingly, aqueous solutions of FeSO_4_·7H_2_O (1 mL, 2 M, in HCl 2 M) and FeCl_3_·6H_2_O (4 mL, 1 M) were simultaneously added to 50 mL of NH_4_OH solution (0.34 M) under mechanical stirring (650 rpm). After 30 min of mixing, the particles were allowed to settle. Following the separation of black sediment by a magnet, the obtained nanoparticles were washed with Milli-Q water several times. At the final step, the particles were centrifuged (3800*g*, 5 min) and redispersed in 50 mL of TMAOH (0.1M) aqueous solution.^28^

For the synthesis of the nanocomposites: PS spheres (100 μL, 100 mg mL^–1^, ∼500 nm in diameter) were diluted into ultrapure water to a final volume of 2 mL and added to 15 mL of PAH (2 mg mL^–1^ in 0.5 M NaCl solution) under soft stirring for 20 min. The excess of PAH was removed by three cycles of centrifugation (3800*g* for 10 min, 20 °C) in ultrapure water. 15 mL of PSS (2 mg mL^–1^ in 0.5 M NaCl solution) was then added, and the procedure was repeated until three layers of alternated polyelectrolytes were obtained. The PS spheres with three layers of polyelectrolytes were then dispersed in 10 mL of ultrapure water, and 2 mL of the presynthesized IONPs (10 mg mL^–1^) was diluted into 5 mL in ultrapure water and mixed with the polyelectrolyte-coated PS solution for 20 min, to obtain iron oxide-coated PS spheres (PS@IONPs). These core–shell nanostructures were included in the final nanocomposites, mixing them for 20 min with a solution containing 0.5 mg of TMV. The final composites were washed by centrifugation and redispersed in ultrapure water.

### Nanocomposite Characterization

Transmission electron microscopy (TEM) images were performed on a JEOL JEM 1010 instrument operating at an acceleration voltage of 100 kV. Scanning electron microscopy (SEM) images were performed on a JEOL JSM-600F. Samples were prepared by dropping a diluted suspension of the nanoparticles onto an ultrathin carbon-coated copper grid or onto silicon oxide substrate, for TEM and SEM, respectively. Samples containing only TMV were counterstained with phosphotungstic acid solution (10 vol % (H_3_PW_12_O_40_)). Magnetic measurements were performed using a vibrating sample magnetometer (VSM) setting in a Physical Property Measurement System (PPMS) from Quantum Design. Hysteresis loops were measured at 300 K up to an external field of 4 or 5 T in powdered samples. The layer-by-layer self-assembly was confirmed by ζ-potential measurements, using a Zetasizer Nanoseries. Raman spectra were collected with a Renishaw InVia Reflex Raman microscope. Experiments were conducted at room temperature using a 532 nm excitation wavelength. The laser beam was focused on the sample by a 50× objective. The experiments were carried out using laser powers of less than 1 mW to avoid overheating. DLS measurements were performed on a Zetasizer Nano ZS (Malvern Instruments Ltd.). UV–vis spectroscopy measurements were carried out using a NanoDrop 2000 spectrophotometer (Thermo Scientific). The magnetic hyperthermia measurements were carried out using a hyperthermia system (MagneTherm, nanoTherics) applying an alternating magnetic field with 12 mT amplitude and 616 kHz frequency. For all experiments, the initial temperature was stabilized before starting the measurement, and the temperature variation was recorded using a thermocouple. Data were plotted and fitted using the corrected slope method and corresponding calculation sheet to finally obtain the heating efficiency SAR value.^29^ Composites from pellet and supernatant upon exposure to the alternating magnetic field were collected for SDS-PAGE analysis.

## Results and Discussion

**Figure 1:**
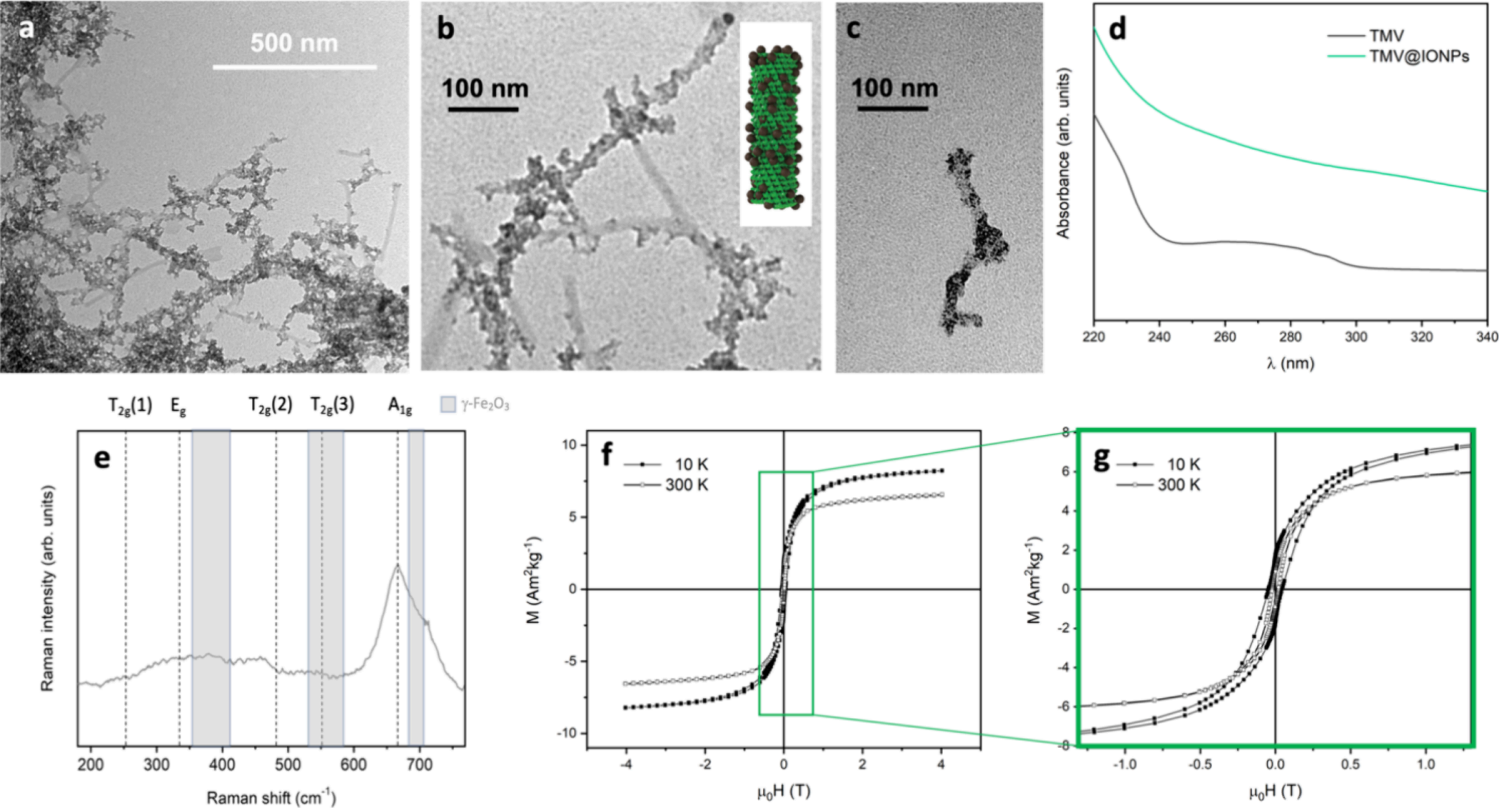
TMV@IONP hybrid nanostructures. (a) Lower- and (b, c) higher-magnification TEM images of the IONPs formed on the TMV ((b) includes a cartoon of the assembly). (d) UV/vis spectra of TMV before (black line) and after (green line) the deposition of the IONPs. (e) Raman spectrum after reducing the iron oxide phase on the TMV with hydrazine at 60 °C, collected using a 532 nm excitation wavelength and laser power <1 mW. (f, g) (Zoomed view) field-dependent magnetization curves (hysteresis loops) of the nanocomposites, recorded at 10 (solid symbols) and 300 K (open symbols).

Prototype nanostructures endorsed with TMV and magnetic IONPs were prepared following two different strategies, with the aim of varying the magnetically induced thermal effects in the virus protein cages. We first considered the synthesis of elongated nanocomposites of TMV coated heterogeneously with magnetic IONPs (TMV@IONPs). Their successful generation was confirmed by TEM (Figure 1a,b, see also Figure S1, in the Supporting Information (SI)). TMV consists of 2130 identical coat protein subunits self-assembled into an elongated nanotube (300 × 18 nm) with a 4 nm hollow channel encapsulating the viral, positive-sense, single-stranded RNA (+ssRNA).^30^ These TMV were purified from *N. benthamiana* plants using established methods^11^ and characterized by UV/vis spectroscopy, chromatography, DLS, and gel electrophoresis, as shown in Figure S2 (in the SI). TMV displayed a characteristic UV/vis spectrum (Figure S2a) with an absorbance peak at 260 nm and a 1.2 (260:280 nm) RNA/protein ratio.^31^ Tyrosine, phenylalanine, and tryptophan amino acids are primarily responsible for the absorption band at 280 nm. The absorption band at shorter wavelengths (220 nm) can be associated with the peptide bonds building the protein backbone and other aromatic groups present in some amino acids. Size exclusion chromatography (Figure S2b) showed the characteristic elution profile with TMV eluting at ∼10 mL and RNA (260 nm) and protein (280 nm) coeluting; disassembled or broken particles were not apparent. Again, the 260:280 nm ratio (RNA/protein) laid at 1.2, which was indicative of pure and intact TMV preparations. While DLS is typically used for spherical nanoparticles, we established DLS for TMV and the hydrodynamic *z*-average size of intact and pure preparations laid at ∼280 nm (Figure S2c). Lastly, purity was confirmed by SDS-PAGE (Figure S 2d), which showed the 17.5 kDa TMV coat protein with no detected impurities. Since these TMV are negatively charged (ζ ∼ −25 mV), they serve as anchors for positive moieties.^32^ Accordingly, we exploited them as nuclei on which magnetic IONPs can be grown, given the hexa-aqueous iron(III) (Fe[H_2_O]_6_^3+^) complexes that formed from the iron(II) sulfate salt and electrostatically interacted with the negative phenolic group of tyrosine. These complexes favor the formation of nanoparticles of an oxyhydroxide phase (by hydrolysis^33^), on the outer shell of TMV, when in the presence of hydrazine. Since oxohydroxides magnetic phases are usually antiferromagnetic (and therefore unable to release heat when under the influence of an alternating magnetic field), we subsequently forced a phase transition of the oxyhydroxide into a ferrimagnetic magnetite/maghemite iron oxide phase, to improve the magnetic properties for hyperthermia. To this end, the nanostructures were suspended again in an aqueous solution while increasing the temperature to enhance the hydrazine reducing strength.^34,35^ This step was performed at 60 °C for 15 min to ensure the phase transition and prevent TMV denaturation. The phase transition is a topotactic transition, implying changes in the crystallographic lattice, and can include the loss of material,^36,37^ justifying the nonuniform distribution of IONPs in the final composites. DLS measurements were performed, in order to check the variation in the hydrodynamic *z*-average size of the TMV before (light green line) and after (dark green line) coated with IONPs (Figure S3, in the SI). The hydrodynamic size registered with the DLS measurements increased from 280 ± 11 nm to 450 ± 28 nm. These differences in the values of hydrodynamic *z*-average size can be justified considering the presence of IONPs on the TMV surface (Figure 1c) and a larger solvation layer due to hydroxide groups present on the surface of the IONPs.

The UV/vis spectra of TMV before and after coated with IONPs were recorded (Figure 1d). Compared to the spectrum of naked TMV (black line), the TMV@IONP nanocomposite (green line) spectrum showed a larger absorption scattering in the whole range. This stems from the larger size attained and the presence of the different iron oxide phases in close contact with the protein capsid, which can also partially damp the TMV absorption, a phenomenon previously reported with chromophore molecules or plasmonic nanoparticles.^38,39^

Raman spectroscopy, widely used to investigate the crystalline structure of transition-metal oxides,^40^ was employed to ascertain the iron oxide phase transition (Figure 1e). Following the hydrazine treatment, a sharp and intense peak at 670 cm^–1^ and a less intense shoulder at 700 cm^–1^ associated with the A_1g_ vibration modes of the spinel structure (magnetite (Fe_3_O_4_) and maghemite (γ-Fe_2_O_3_) A_1g_ modes, respectively),^41,42^ confirmed the formation of magnetic nanoparticles with these two different iron oxide phases on TMV. The magnetic behavior displayed in Figure 1f,g also confirmed the crystalline lattice crossover registered by Raman spectroscopy, since the characteristic ferrimagnetic field-dependent magnetization curves were observed. Nevertheless, the fact that the magnetization does not saturate but keeps increasing with the field implies the presence of an antiferromagnetic magnetic phase (likely the oxyhydroxide lepidocrocite γ-FeOOH, although not detected by Raman spectroscopy). Figure 1g includes both curves at 10 and 300 K and at low field, to appreciate the superparamagnetic behavior characteristic of very small ferrimagnetic nanoparticles, with a very small value of coercivity at room temperature. Low values of magnetization saturation (6.5 Am^2^ kg^–1^ at 300 K and 8 Am^2^ kg^–1^ at 10 K) stemmed from the low percentage of ferrimagnetic material in the final hybrid composites,^43^ and the small coercivity at 10 K can be justified in terms of an effective magnetocrystalline anisotropy of these magnetic nanoparticles because of the different iron oxide phases.

**Figure 2:**
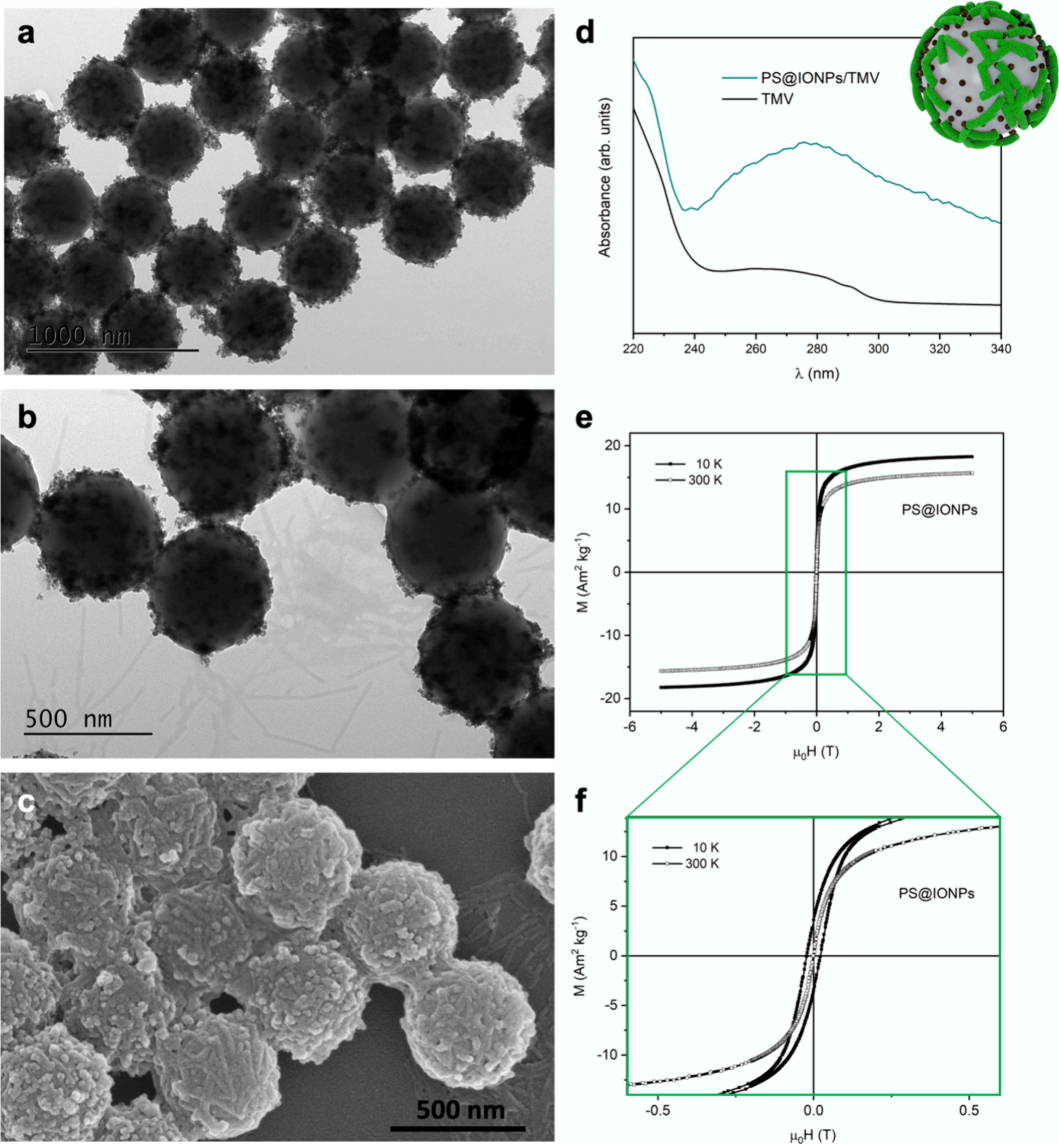
PS@IONPs/TMV hybrid nanostructures. TEM images of the PS@IONP composites before (a) and after (b) depositing the TMV. (c) Representative SEM image of the PS@IONPs/TMV hybrid nanostructures. (d) UV/vis spectra of TMV (as reference, black line) and of the PS@IONPs/TMV composites (green line) (inset: cartoon of the hybrid nanostructure). (e, f) (Zoomed view) field-dependent magnetization curves (hysteresis loops) of the composites, recorded at 10 (solid symbols) and 300 K (open symbols).

For the second prototype, we pursued the use of PS spheres (which are considered biocompatible up to a threshold concentration^23,44^) for the deposition of presynthesized IONPs and TMV via layer-by-layer self-assembly, based on a previously established protocol that starts with the deposition of three layers of polyelectrolytes (PAH/PSS/PAH; the UV–vis spectra are included in Figure S4a, in the SI). The deposition of this polyelectrolyte multilayer ensures a homogeneously distributed surface charge,^45^ to obtain PS@IONPs/TMV (Figure 2). The IONPs employed in this case were presynthesized using the Massart coprecipitation method^27^ and measured on average 11.0 */ 1.0 nm (in diameter, log-normal fit) (Figure S5a and inset, in the SI). Figure S5b (in the SI) shows the Raman spectra of the presynthesized IONPs before and after treated with TMAOH. Both Raman spectra included show the presence of the intense A_1g_ band at 670 cm^–1^ and two less intense, at 300 and 530 cm^–1^, associated with Fe_3_O_4_ (areas shaded in dark blue). The shoulder at 700 cm^–1^ and two subtle bands at 300 and 460 cm^–1^ (areas shaded in light blue), which become more intense after fixing the TMAOH molecules, confirm the presence of maghemite (γ-Fe_2_O_3_) as a second phase.

Once synthesized and stabilized in aqueous solution, these IONPs were subsequently fixed on the surface of 500 nm PS spheres taking advantage of electrostatic interactions between the previously deposited polyelectrolyte layers and the nanoparticles themselves. To maximize the magnetic response for hyperthermia of these final hybrids of PS spheres coated with IONPs and TMV, an optimization process previously developed in our group, was considered, tuning the amount of IONPs per PS sphere.^44^Figure 2a includes a TEM image of the PS spheres decorated with these presynthesized IONPs homogeneously distributed. These PS@IONPs composites were further decorated with TMV using the remaining positively charged functional groups of the outer polyelectrolyte PAH layer initially deposited on the PS spheres. The successful deposition of the TMV was confirmed by electron microscopy (Figure 2b (TEM) and Figure 2c (SEM)). Although the TMV on the PS@IONPs in Figure 2b are not clearly visible, their presence results in a blurrier contrast in this TEM image compared to the TEM image in Figure 2a. Furthermore, despite the large scattering of the 500 nm PS spheres and the PS@IONPs (see Figure S4b in the SI), the UV/vis spectrum of the PS spheres coated with IONPs and TMV shows a large band that can be associated with the 260 and 280 nm absorption bands of the TMV (Figure 2d). Absorption associated with the polyelectrolytes can be excluded, since they are screened because of the PS and IONP scattering (see Figure S4b in the SI). Figure 2e includes the field-dependent magnetization curves of the hybrid composites, at 10 and 300 K (at low field in Figure 2f, to appreciate the superparamagnetic behavior of the IONPs), showing relatively small values of magnetization saturation (15 Am^2^ kg^–1^ at 300 K and 18 Am^2^ kg^–1^ at 10 K) due to the low percentage of ferrimagnetic material in the final hybrid composites.^43^

Next, we compared the capacity of the magnetic nanoparticles in the two prototypes of hybrid nanocomposites (TMV@IONPs and PS@IONPs/TMV) to deliver heat via magnetic induction, that is, under the influence of an alternating magnetic field (AMF). For that, (1) the time scale of one cycle of the AMF has to be shorter than the nanoparticle response via thermal relaxation mechanisms (Neel and Brownian) and (2) the magnetic field strength must be sufficient to overpass the effective magnetic anisotropy barrier of the nanoparticles.^46^ Of the two possible methods (AC magnetometry and calorimetric measurements) commonly used to determine the heating performance of magnetic particles in solution, we have employed the later because it accounts for measuring the increase of the temperature of the magnetic colloid in aqueous solution over time.^47^

Accordingly, both magnetic virus-based assemblies were dispersed in aqueous solution and subjected to an AMF of 12 mT amplitude and 616 kHz frequency, to study the thermal effects on the protein cages, in view of the different magnetic phase and position with respect to the virus of the heating sources (the magnetic nanoparticles). The product *H*_0_*f* (amplitude per frequency of the magnetic field employed) was kept below the physiological limit required for potential *in vivo* experiments (*H*_0_*f* ≤ 5 × 10^9^ Am^–1^ s^–1^).^48,49^ The obtained data was processed and fitted according to the temperature evolution profile, taking into consideration nonadiabatic conditions,^47^ to finally evaluate the efficiency of the magnetic nanoparticles to deliver heat, in terms of the SAR (specific adsorption rate) value:
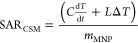
where *C* is the specific heat capacity of water (4184 J·kg^–1^·K^–1^), *m*_MNP_ (g) is the mass of magnetic material employed, d*T*/d*t* is the temperature variation within the time frame of the recording, *L* is heat losses, and Δ*T* is the average temperature difference between the sample and the baseline. For the calculation of these SAR values, 5 min of exposure time was considered during the temperature increase evaluation, to minimize heat exchange/loses. The obtained temperature kinetics of the aqueous solutions of the two types of hybrids are reported in Figure S6 (in the SI). SAR values of 21.87 (±1.10) and 5.69 (±0.104) W g^–1^ for the distribution of IONPs in the TMV@IONPs and in the PS@IONPs/TMV hybrids, respectively, were subsequently estimated. For this calculation, assessed concentrations of Fe from the IONPs of ∼0.8 and ∼5 mg/mL, respectively, were employed. The different heat delivery performances of the two prototypes can be attributed to two main factors: (1) the magnetic particles employed, which are made of different magnetic iron oxides with distinct size and shape, and (2) their spatial distribution as heat sources (individually fixed, aggregated, or in a linear assembly) in the final hybrid structure.^50−55^ Theoretical studies have demonstrated that the spatial distribution of magnetic nanoparticles on a surface implies different heating efficiencies.^56^ For instance, while a linear noninteracting arrangement of nanoparticles had a SAR value of 850 W g^–1^ (*f* = 1000 kHz, *H*_0_ = 26 mT), the corresponding random distribution displayed a SAR value of 300 W g^–1^ and the random agglomeration of the same particles resulted in values below 100 W g^–^^1^, which can explain the low values of SAR registered in these composites, with the magnetic nanoparticles largely interacting. Nevertheless, we need to consider that the heat generated by the IONPs in both cases serves two purposes: (a) to disassemble and potentially denature the proteins of the TMV and (b) to raise the temperature of the solution. However, we only account for the temperature increase when calculating the SAR values in terms of the recorded temperature kinetics. This implies that the heat associated with the disassembly of the protein cages is not factored into this calculation. To illustrate this, Figure S7 includes a graph that depicts the temperature increase of the IONPs on the PS, with and without the virus, showing a greater temperature rise in the latter scenario. This indicates indeed that some of the heat generated by the magnetic nanoparticles is utilized in the process of disassembling the protein cages.

**Figure 3:**
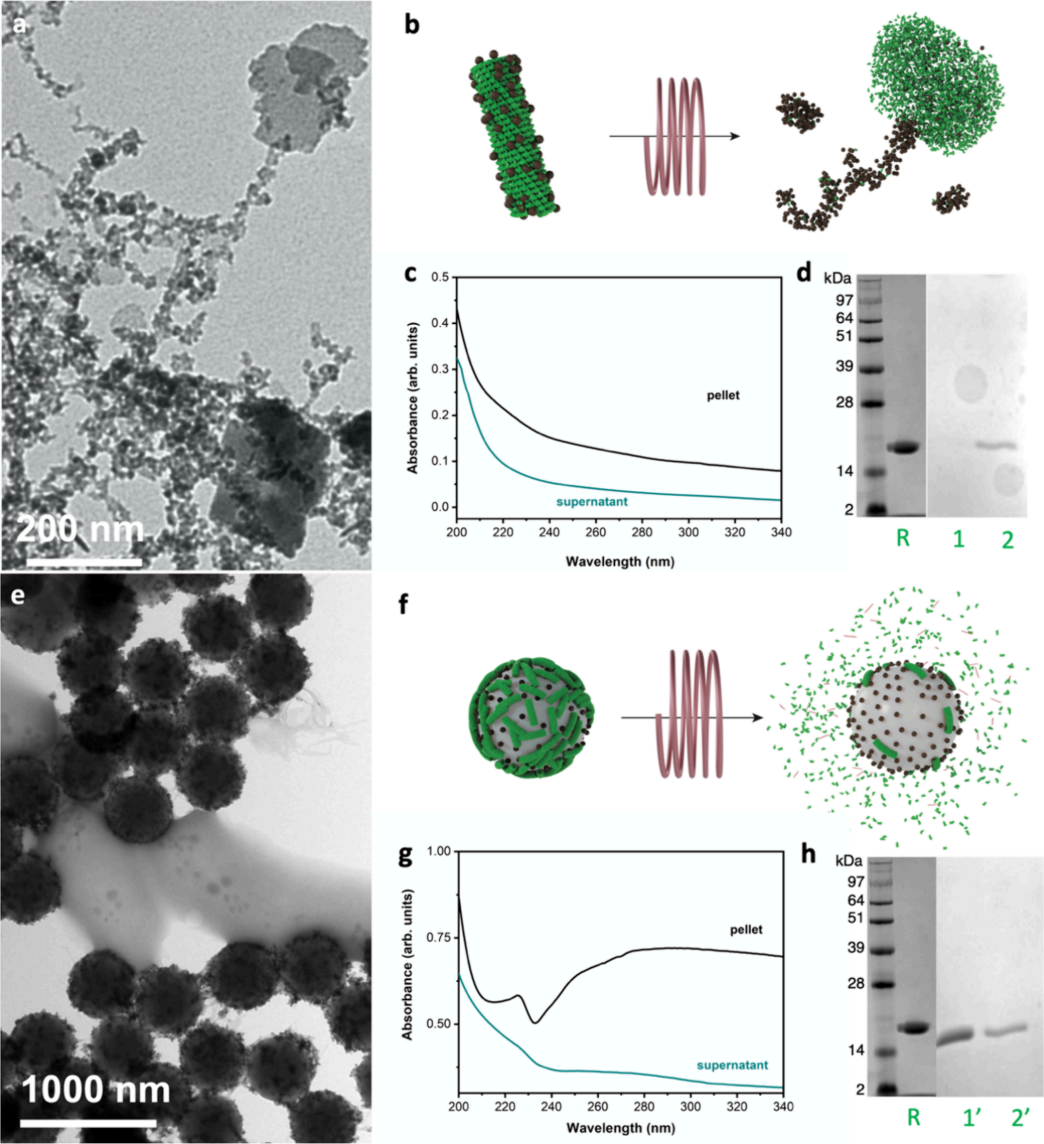
Evaluation of the hybrids integrity upon hyperthermia. (a) TEM image, (b) scheme of the hyperthermia process, (c) UV/vis spectra, and (d) SDS-PAGE of the TMV@IONPs after the heat release, in the pellet (lane 1) or in the supernatant (lane 2) using the initial TMV as reference (lane R). (e) TEM image, (f) scheme of the hyperthermia process, (g) UV/vis spectra, and (h) SDS-PAGE of the PS@IONPs/TMV after the heat release, in the pellet (lane 1′) or in the supernatant (lane 2′) using the initial TMV as reference (lane R).

Finally, the integrity of the protein cages in the TMV@IONPs and PS@IONPs/TMV hybrids under the influence of the heat generated by the IONPs was evaluated. For that, electron microscopy, UV/vis spectroscopy, and SDS-PAGE (using the 17.5 kDa TMV CP as reference), following exposure of the hybrids to the AMF (616 kHz, 12 mT), were employed (Figure 3). The local heat delivered by the two types of IONPs had a profound effect on the integrity of the TMV and consequently on the morphology of the prototypes. Within the TMV@IONP formulation, the proteins of the TMV transitioned from a cylindrical to an aggregated shape (Figure 3a,b (sketch of the process)). This heat-induced morphological change of TMV has been previously reported.^57,58^ The individual 2130 coat proteins self-assembling into the rod-shaped TMV can form spherical structures with tunable diameters depending on the initial concentration of coat proteins and the temperature of the process.^57^ Temperatures between 94 and 98 °C were reported to induce the complete conversion of TMV rods into spheres. Using magnetic heating, the thermal transition occurred at a much smaller concentration of TMV (ca. 0.55 mg mL^–1^) and at a lower temperature range (∼21.0–21.6 °C). Nevertheless, the local temperature at the surface of the magnetic nanoparticles acting as heat sources is likely much higher.^59,60^ The average size of these protein agglomerates was ∼200 nm (Figure 3a, TEM image). Based on the volume of rod-shaped TMV, spherical agglomerates of ∼50 nm in diameter can be attained from each TMV rod.^57^ Coat protein units from more than one TMV rod were therefore required to attain these larger protein aggregates. Furthermore, the magnetic nanoparticles initially fixed on the protein cages become free in solution and agglomerated when observed in the TEM grid (Figure 3a, TEM image).

After exposure to the AMF, the UV/vis spectra of the TMV@IONP nanostructures were obtained from both the residual pellet (black line) and the supernatant (green line) following centrifugation (Figure 3c). The resulting UV/vis absorptions displayed no signs of the presence of small or large protein fragments, amino acids, or RNA. For a more detailed analysis of the disassembly process triggered by the thermal effects, SDS-PAGE electrophoresis analysis was also employed. Figure 3d includes the electrophoretic mobility of both the pellet and the supernatant obtained after the TMV@IONP exposure to the AMF. To identify the proteins left in solution, TMV coat proteins (CP) (without any treatment) were used as reference (lane R). In the pellet containing the 200 nm agglomerates of proteins, no individual 17 kDa TMV coat proteins were detected (lane 1), while a small fraction of TMV coat proteins were present in the supernatant (lane 2). These results indicate that the heating was powerful enough to disassemble the protein cages of the virus to form the aggregates but also disassembled the coat proteins into much smaller pieces or fragments, no longer detectable by SDS-PAGE.

When it comes to the PS@IONPs/TMV formulation after heat treatment, TMV was partially peeled off and disassembled (Figure 3e,f (sketch of the process)), producing some agglomeration of protein-based organic material surrounding the PS spheres still coated with the IONPs and a few TMV, as shown in the TEM image. The corresponding UV/vis spectra (Figure 3g) of these hybrids after exposure to the AMF indicated the release of small protein units in the supernatant (green line). The spectrum registered from the pellet after centrifugation indicated the presence of proteins from both the disassembled and intact virus capsids, with some relative maxima that can be associated with the simultaneous presence of polyelectrolytes, protein and nuclei acid, which overlap, and the scattering because of the PS that systematically distorts the spectrum (*vide infra*). Along these lines, the SDS-PAGE electrophoresis analysis (Figure 3h) demonstrated the presence of TMV coat proteins in both the pellet (lane 1) and supernatant (lane 2), reflecting that the heat release just disintegrated a fraction of the initial TMV, likely those closer to the heating sources (the magnetic nanoparticles), and leaving the rest intact.

**Figure 4:**
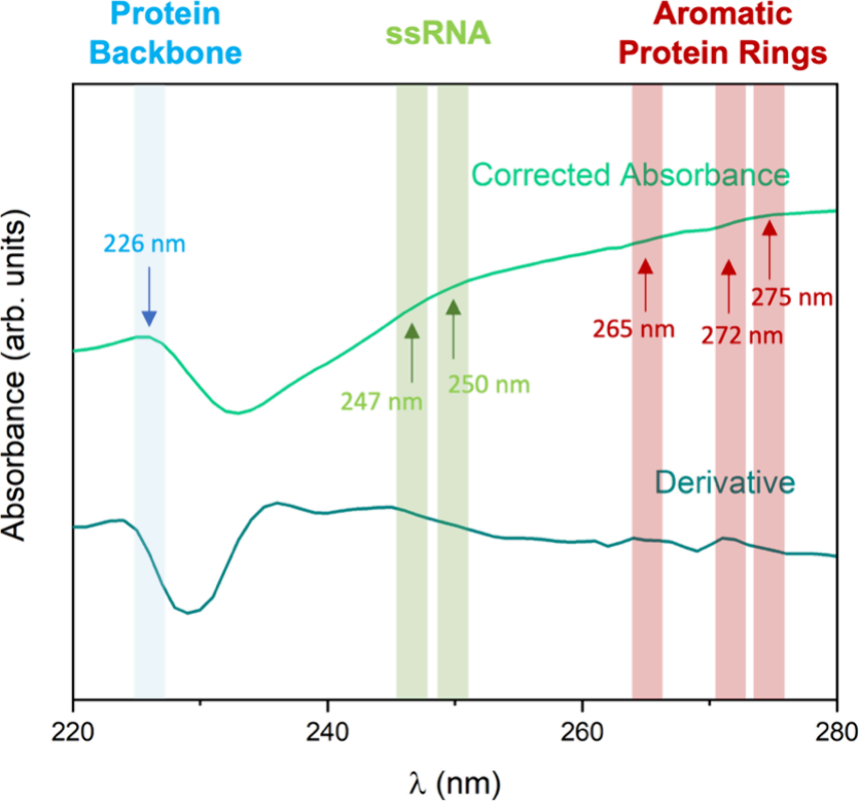
Zero-order and first-order derivative UV/vis (corrected) spectrum of the PS@IONPs/TMV after the heat release, with a local maximum at 226 nm (shadowed in blue and associated with the protein backbone) and shoulders at 247 and 250 nm (shadowed in green and associated with nuclei acids) and at 265, 272, and 275 nm (shadowed in pink and associated with amino acids).

Since an RNA/protein (260/280 nm) ratio of 1.2 in a UV/vis spectrum is indicative of intact TMV in solution,^61,62^ larger or lower values of this ratio can imply partial or total degradation of the viruses, leading to the presence of just proteins (260/280 ratio = 0.65) or just free RNA (260/280 ratio = 2).^63^ The spectrum of the PS@IONPs/TMV hybrids in the pellet after the heat treatment (Figure 3g, black line) showed a (260/280) ratio of 0.92 (±0.01), which, as mentioned, can be associated with the presence of intact and degraded TMV, in agreement with the TEM analysis (Figure 3e). Still, since this spectrum absorbance is influenced and even distorted because of the PS@IONPs' large scattering (see Figure S4b), to have a more accurate interpretation, we have considered a method that corrects the light scattering and ponders the presence of both protein and nuclei acid.^64^Figure S8 in the SI includes the comparison of this spectrum before and after applying this method, by which a partial contribution of light scattering can be subtracted. Once the absorbance spectrum was corrected, we further proceeded considering its derivative (d*A*/dλ), as shown in Figure 4. The transformation of a UV/vis spectrum to its first derivative yields a more complex profile without increasing the intrinsic information content and offers a qualitative analysis for positive identification of trace levels of a component in the presence of a strongly absorbing matrix.^65^ This is illustrated in the first derivative of the corrected absorbance spectrum (Figure 4), whose resolution is improved. Besides the maximum (shadowed in blue, at 226 nm) associated with the protein backbone, the first derivative permits to identify the presence of shoulders (shadowed in green at 247 and 250 nm and in pink at 265, 272, and 275 nm), associated with the guanine and adenine nucleobases present in the RNA, and to the phenylalanine, tryptophan, and tyrosine amino acids, respectively, confirming the disassembly of the protein cages and the release of RNA.

Upon exposure to the AMF, the two types of hybrids were distinctively impacted by the local increase of temperature, either because of the different heating efficiency of the IONPs reflected in the estimated SAR values or because of the different relative positions of the heating sources with respect to the virus. In this regard, although release of the RNA from the TMV@IONPs hybrids is likely to have happened, its likely instability and rapid degradation because of the high temperature prevented us from detecting it by UV/vis. Alternatively, the detection of traces of guanine and adenine confirmed the release of the RNA in the case of PS@IONPs/TMV hybrids. With that into account, we can consider slow and fast release mechanisms, in a similar way as those simulated based on the nature and interactions of the capsid proteins within protein cages.^21,66^ If capsids were cracked open, drug and/or genome would be rapidly released due to the fast dynamics and the stochastic (with a random probability distribution) nature of the release process. This could here be the case considering the heating sources directly attached to the proteins, forcing the capsid to break apart into fragments, and subsequently reassembled to form the protein aggregates observed when considering the TMV@IONP hybrids. Otherwise, we can assume a much slower disassembly mechanism in the case of the PS@IONPs/TMV, given that the heating sources are close but not in direct contact with the protein capsids and are therefore able to disassemble only a fraction of the TMV from which small fragments of protein, amino acids, and traces of nucleobases were detected.

## Conclusions

In summary, two assemblies of magnetic nanoparticles and tobacco mosaic virus were achieved. The hybrid nanostructures were characterized in terms of morphology, magnetic phase, and spatial distribution of the magnetic material relative to the virus. The programmable and magnetically induced hyperthermia resulted in the disassembly of all, or a fraction of the protein cages present in the nanocomposites. The thermal effects were more pronounced in the case of hybrid nanocomposites of tobacco mosaic viruses coated with the iron oxide nanoparticles (TMV@IONPs), because of the direct proximity of the heating sources and the protein cages, inducing the formation of protein aggregates. The thermal effects were less pronounced when not in direct contact (in the PS@IONPs/TMV), resulting in the disassembly of the coat protein building units of some of the viruses. In both cases, the disassembly of the protein cages led to the release of the RNA, small proteins fragments, and amino acids dispersed in the final solution, but likely clamped in the final protein spheroids or degraded in the final solution, and only traces of nucleobases and amino acids were detected when employing the less effective heating process.

This work highlights the potential of combining plant viruses with magnetic nanoparticles to revolutionize targeted drug and gene delivery with a programmable disassembly of the protein capsids. However, there are several challenges and areas of optimization that need further exploration, such as accurate loading and release to minimize off-target effects, heat delivery and disassembly without damaging the cargo and surroundings, versatility in terms of both viral structures and types of magnetic nanoparticles, and magnetic guidance for a targeted delivery of drugs and genetic material. In conclusion, these proof-of-concept prototypes provide a starting point for further research and development, with the potential to revolutionize therapeutic delivery methods by enhancing precision, reducing side effects, and maximizing therapeutic efficacy.

## Supporting Information Available

Low- and high-magnification TEM images of the TMV@IONPs, TMV characterization, DLS measurements, UV–vis spectra of polyelectrolytes, IONP characterization, hyperthermia experiments, and UV–vis spectra with and without corrected absorbance (PDF)
