# Supplementary material for: Magnetically Induced Thermal Effects on Tobacco Mosaic Virus-Based Nanocomposites for a Programmed Disassembly of Protein Cages
Source: ACS Appl Bio Mater. 2024 Jun 27;7(7):4804–14. doi: 10.1021/acsabm.4c00634 (PMC11253087; doi:10.1021/acsabm.4c00634)
Supplement: Supplementary file 1 — mt4c00634_si_001.pdf [file mt4c00634_si_001.pdf]

Supporting information

Magnetically induced Thermal Effects on Tobacco Mosaic Virus-based Nanocomposites for a Programmed Disassembly of Protein Cages

Ecem Tiryaki,[1] Carla Álvarez-Leirós,[1] Julia N. Majcherkiewicz,[1]

Paul L. Chariou,[2] Melodie Maceira-Campos,[1] Gustavo Bodelón,[1,3]

Nicole F. Steinmetz,[2,4,5,6,7] Verónica Salgueiriño[1,8]**

[1] CINBIO, Universidade de Vigo, 36310 Vigo, Spain

[2] Department of Bioengineering, University of California, La Jolla, San Diego (CA), USA

[3] Departamento de Biología Funcional y Ciencias de la Salud, Universidade de Vigo, 36310, Vigo, Spain

[4] Department of NanoEngineering, University of California, La Jolla, San Diego (CA), USA

[5] Department of Radiology, University of California, La Jolla, San Diego (CA), USA

[6] Center for Nano-ImmunoEngineering, University of California, La Jolla, San Diego (CA), USA

[7] Institute for Materials Discovery and Design, University of California, La Jolla, San Diego (CA), USA

[8] Departamento de Física Aplicada, Universidade de Vigo, 36310 Vigo, Spain

Corresponding authors:

Nicole F. Steinmetz (nsteinmetz@ucsd.edu)

Verónica Salgueiriño (vsalgue@uvigo.gal)

KEYWORDS. Tobacco Mosaic Virus, Magnetic Nanoparticles, Nanocomposites, Magnetic Hyperthermia, SAR Values, Protein Cage Disassembly, Gene Delivery.

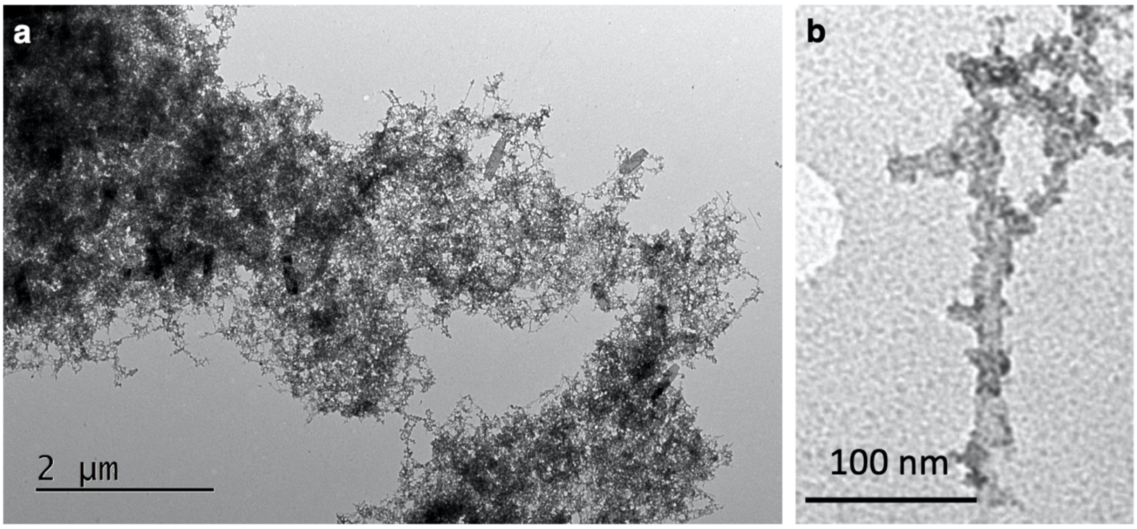

Figure S1. Low (a) and high (b) magnification TEM images of the TMV@IONPs synthesized.

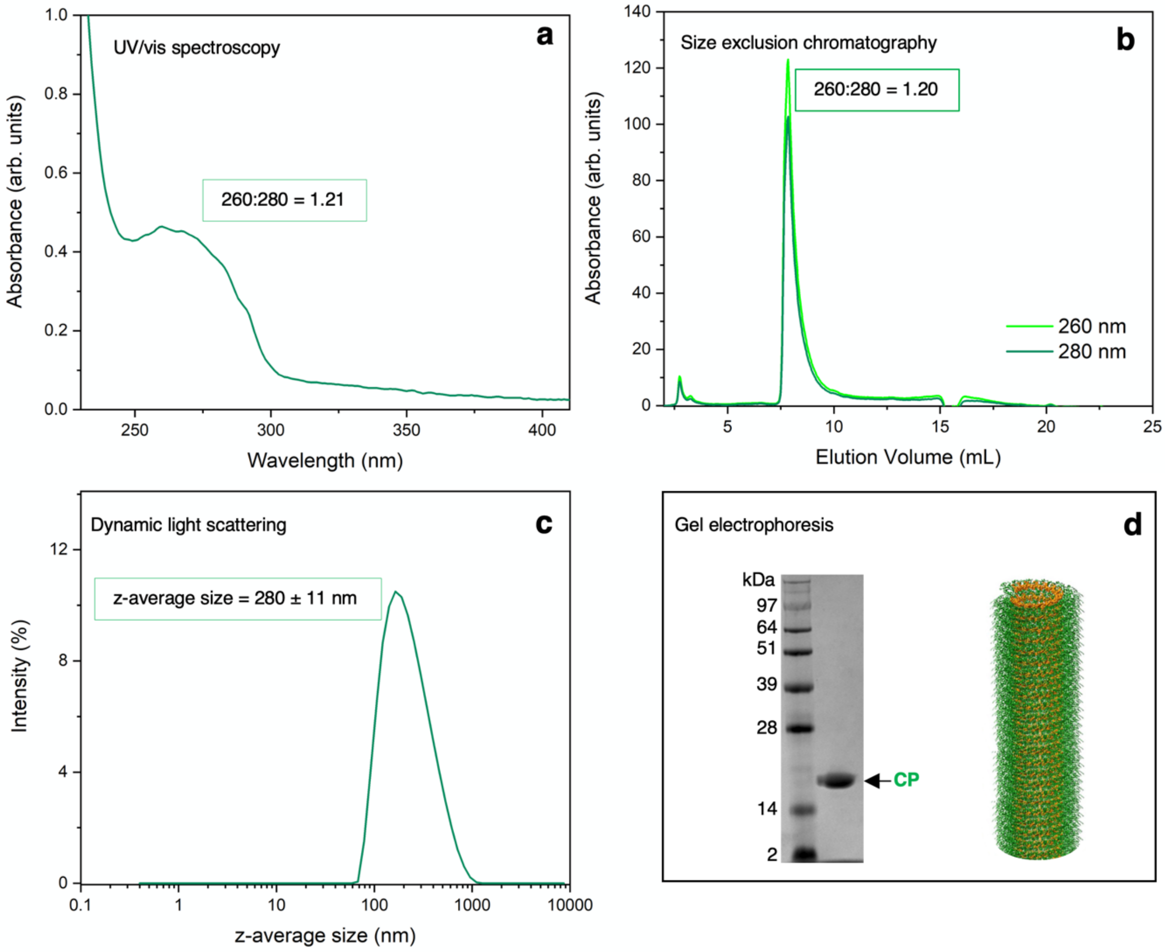

Figure S2. TMV characterization. a) UV-vis spectrum of TMV. **b**) Corresponding size exclusion Chromatography (SEC). The absorbance ratio of RNA (260 nm) to protein (280 nm) is displayed in the insets. **c**) TMV z-average size distribution obtained by dynamic light scattering (DLS). The z-average size was calculated as the weighted mean of the intensity distribution. **d**) Representative denaturing SDS-PAGE gel captured under white light and stained for proteins using Coomassie Brilliant blue in the presence of the SeeBlue Plus2 ladder. The location of TMV coat proteins (CP) is indicated by an arrow.

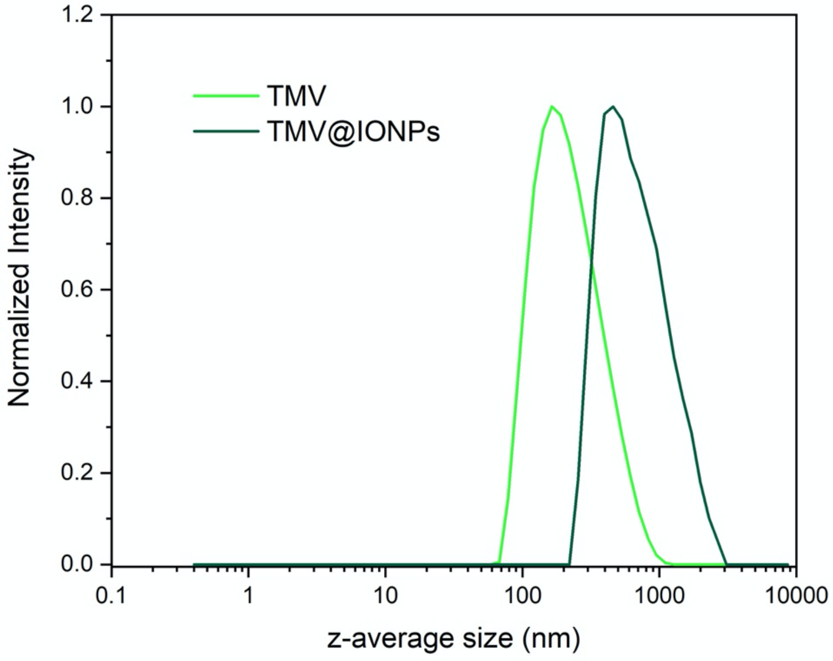

Figure S3. DLS measurements reflecting the z-average size of the TMV before and after coated with IONPs.

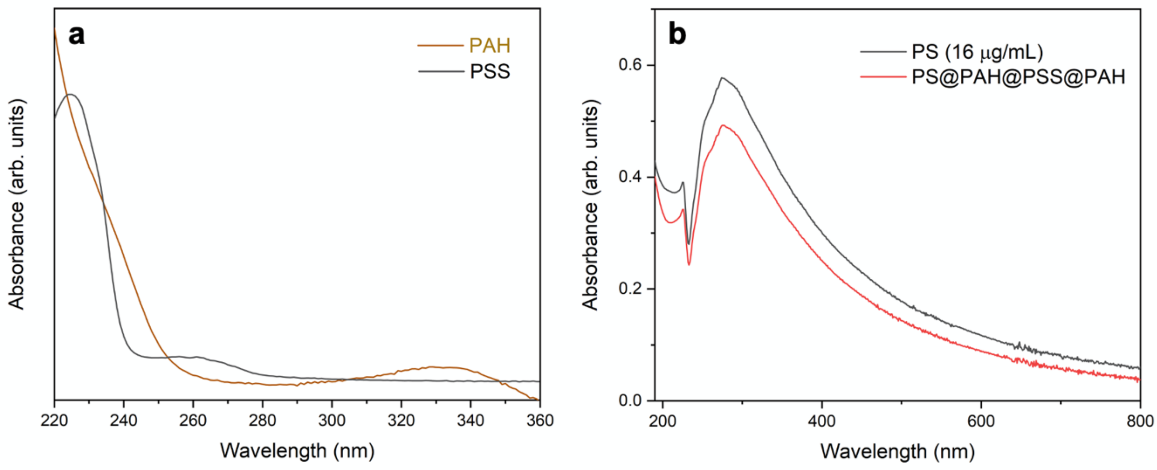

Figure S4. UV-vis spectra of the polyelectrolytes forming part of the PS@IONPs/TMV nanocomposites. a) UV-vis spectra of PAH and PSS polyelectrolyte in solution (1 and 0.1 mg/mL, respectively) and b) UV-vis spectra of the PS spheres before and after coated with three layers of polyelectrolytes (PAH/PSS/PAH).

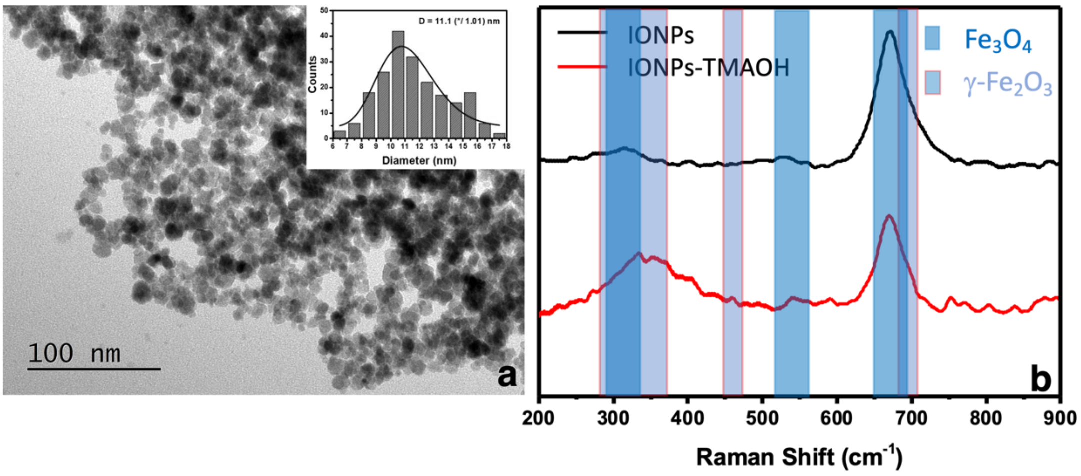

Figure S5. Pre-synthesized IONPs characterization. a) representative TEM image (inset: size distribution analysis fitted to a log-normal curve) and **b)** Raman spectra before and after treated with TMAOH (areas shadowed in dark and light blue indicate the position of the vibration modes of magnetite (Fe_3O_4) and maghemite (γ-Fe_2O_3)).

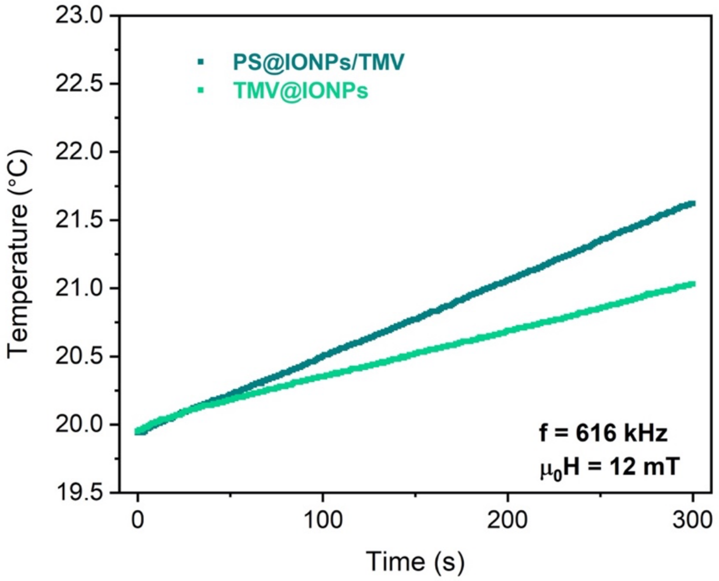

Figure S6. Hyperthermia experiments. Temperature kinetics of the water dispersions of the two hybrid nanocomposites (TMV@IONPs (light green symbols) and PS@IONPs/TMV (dark green symbols) during the exposure to the AMF field (12 mT amplitude and 616 kHz fixed frequency), for the determination of the SAR values. The starting temperature was ~20 ºC for both experiments.

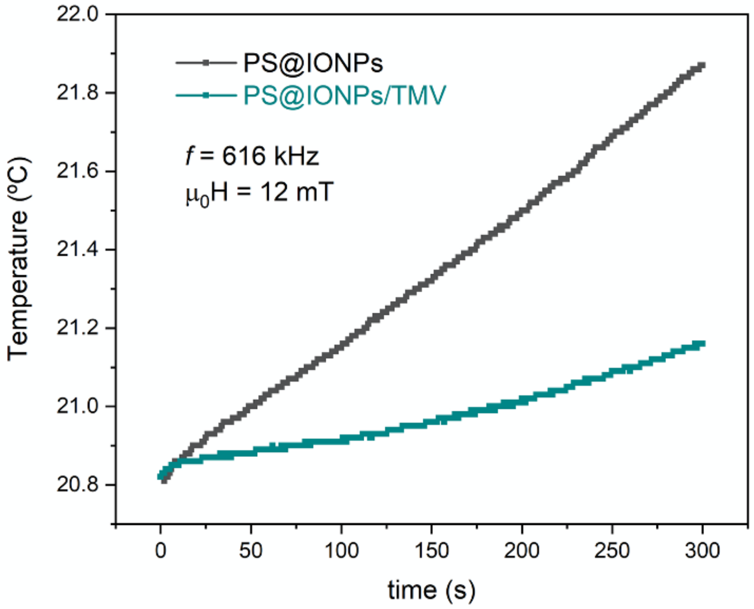

Figure S7. Hyperthermia experiments. Temperature kinetics of the water dispersions of the PS@IONPs, with (green symbols) and without (black symbols) the TMV fixed on the surface during the exposure to the AMF field (12 mT amplitude and 616 kHz fixed frequency). The starting temperature was ~20.8 ºC for both experiments.

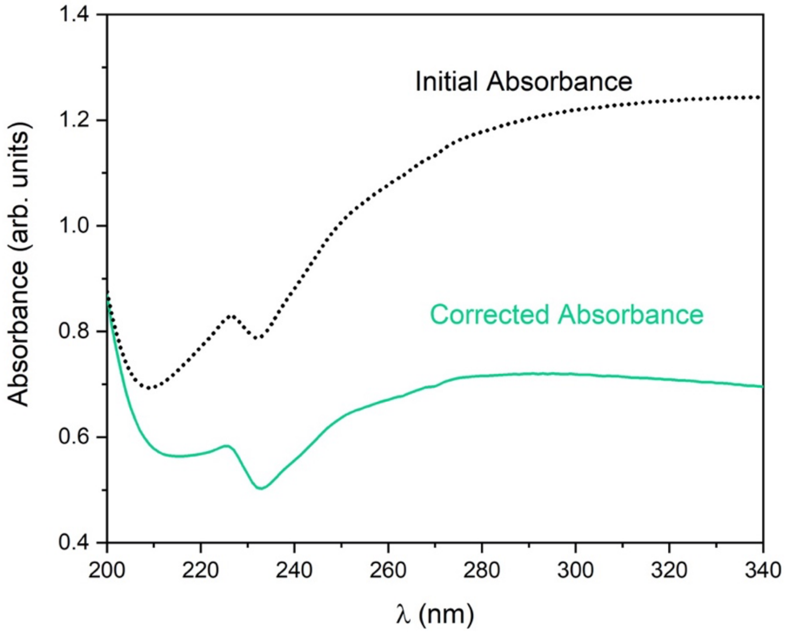

Figure S8. Experimental and corrected absorbance spectra of the PS@IONPs/TMV nanocomposites upon exposure to the AMF.
